# Sociodemographic, clinical and organisational factors associated with delayed hospital discharges: a cross-sectional study

**DOI:** 10.1186/1472-6963-14-128

**Published:** 2014-03-15

**Authors:** Jacopo Lenzi, Maria Mongardi, Paola Rucci, Eugenio Di Ruscio, Maria Vizioli, Concetta Randazzo, Elena Toschi, Tiziano Carradori, Maria Pia Fantini

**Affiliations:** 1 Department of Biomedical and Neuromotor Sciences, Alma Mater Studiorum, University of Bologna, Via San Giacomo 12, Bologna 40126, Italy; 2 Regional Agency for Health and Social Care of Emilia-Romagna, Bologna, Italy

**Keywords:** Delayed discharges, Patient flow, Survey, Intermediate care, Multilevel analysis

## Abstract

**Background:**

Evidence from studies conducted in Western countries indicates that a significant proportion of hospital beds are occupied by patients who experience a delayed hospital discharge (DHD). However, evidence about this topic is lacking in Italy, and little is known on the patients’ and organisational characteristics that influence DHDs. Therefore, we carried out a survey in all the hospitals of a Northern Italian region to analyse the prevalence and the determinants of DHD.

**Methods:**

A cross-sectional study was carried out during an index period of 15 days in 256 operative units in Emilia-Romagna, a Northern Italian region with 4.4 million inhabitants, to identify patients medically fit for discharge but still hospitalised. The characteristics of these patients (*n* = 510) were compared with all the other patients (*n* = 5,815) hospitalised in the same operative units during the index period using multilevel logistic regression models.

**Results:**

The one-day prevalence of DHD was 8.1%. More than half of DHD patients (52.7%) waited to access long-term/rehabilitation units or residential care homes, 16.7% experienced a delay for family-related reasons, and 14.5% were waiting to be admitted to other rehabilitation services. Among DHD patients hospitalised in long-term/rehabilitation units, 45.3% were waiting to be transferred to residential care homes. Patients’ characteristics associated with a higher likelihood of DHD in multilevel logistic regression were older age, provision of intensive care, a diagnosis of dementia, tumours or femoral/shoulder fractures, and a number of comorbidities. Patients hospitalised in long-term/rehabilitation units, as well as in orthopaedics/traumatology units, were significantly more likely to have a DHD compared with patients hospitalised in general surgery units. Moreover, compared with Local Health Authority Hospitals, being hospitalised in Hospital Trusts was associated with a higher likelihood of DHD.

**Conclusions:**

Although the prevalence of DHD in the present study is markedly lower than that reported in the literature, we submit that the DHD problem should be addressed with major organisational innovations, with a special focus on the ageing of the population and epidemiological trends. Organisational changes imply new ways of managing emerging clusters of patients whose needs are not efficiently or effectively met by traditional organisation models and services.

## Background

Studies conducted in various countries show that a significant proportion of hospital beds are occupied by patients that experience delayed discharge because they cannot be transferred to rehabilitation/residential facilities or moved back home [1-3]. In a recent systematic review, the percentage of inappropriate use of acute care beds has been found to range between 15% and 50%, with differences due to the various contexts of care, definitions and methodologies adopted [4]. Prolonged hospital stays may increase the risk of infections and other iatrogenic complications, worsen the patient’s quality of life especially in the elderly [5], and imply a waste of economic and human resources [6].

Italy has a National Healthcare System (NHS) that provides uniform and comprehensive care. Under the Italian Constitution and its recent modification (2001), the State has exclusive power to set the “essential levels of care” for all residents throughout the country, while the twenty regions have responsibility for the organisation and administration of publicly financed healthcare. NHS is financed by national and regional taxes (97%) and patient’s copayments; according to the State General Accounting Department, public healthcare expenditure in 2011 was 112 billion euro in current prices, 7.1% of gross domestic product [7].

The hospital sector has long dominated the healthcare system in Italy. Inpatient care still accounts for 48% of total public healthcare expenditure nationally and in some regions for almost 54%. Official policy for many years was to reduce NHS bed capacity (the national standard fell from 11 to 3.7 beds per 1,000 inhabitants between 1975 and 2012) and to fund public and private contracted hospitals on a full cost basis.

Primary healthcare is provided by general practitioners and family paediatricians paid by capitation, who assess the needs of patients, order diagnostic procedures, prescribe drugs, and refer patients to specialists and hospitals [8]. In this sense, they act as “gatekeepers” for the system and ensure continuity of care to patients with chronic diseases.

Recently, the Italian government has been taking initiatives to re-allot diminished economic resources for the healthcare system; thus, it is fundamental to investigate and remove inefficiencies in order to prevent indiscriminate cuts of goods and services. Since hospital admissions account for a very large part of health expenditure [9], an improvement of bed management for acute care is needed.

Delayed discharges may be regarded as an indicator of inefficient use of hospital beds. Factors associated with delayed hospital discharges have been attributed to patients’ demographic, medical, family and dependency characteristics, as well as organisational and administrative processes [10]. A survey carried out in an inner-London health district revealed that most delayed patients were aged and hospitalised in geriatrics (43%), medical (27%) and surgical units (6%) [11]. Elderly patients with comorbidities and complex healthcare needs are indeed more prone to receive long-term care, and therefore more likely to suffer a delay in being discharged from hospital. In Italy, a recent pragmatic trial carried out in twelve medical wards chosen to have the longest lengths of stay, showed that medical staff involvement and audit/feedback activity reduced by 16% the proportion of patients with a prolonged hospital stay not justified by clinical conditions [12]. Among organisational factors of delay, some studies emphasise the role of both hospital and primary care processes [13,14]. Hospital discharge is indeed appropriate when the patient does not exhibit evolving clinical symptoms and is assisted after the acute stage of illness through processes that involve different professionals and both health and social facilities [15,16].

Nonetheless, little is known on the patients’ and organisational characteristics that influence delayed discharges, and there is a lack of evidence about this topic in Italy. We thus carried out a survey over an index period in all the hospitals operating in Emilia-Romagna region (Northern Italy) in order to inform regional stakeholders about the prevalence and the determinants of delayed discharges. To our knowledge, no previous study on delayed hospital discharges and their predictors has so far comprised a large number of units from different medical disciplines.

## Methods

The survey was carried out at 256 operative units of general surgery, geriatrics, internal medicine, orthopaedics/traumatology, and long-term/rehabilitation, that comprise 91.4% of all the units of the same disciplines with acute hospital beds^a^ operating in Emilia-Romagna region (4.4 million inhabitants, 42% aged >50 years). This cross-sectional study involved 58 public hospitals; of these, 52 were run by Local Health Authorities (*Aziende Sanitarie Locali*),^b^ while 6 were Hospital Trusts (*Aziende Ospedaliere*),^c^ public enterprises with a legal status broadly similar to that of the British trust hospitals [8].

The Regional Work Team on delayed hospital discharges, including one representative for each Local Health Authority or Hospital Trust, designed the survey and trained to the study procedures one physician and one nurse designated for each hospital in a one-day session. More specifically, physicians and nurses were trained to identify delayed patients based on the definition proposed by Bryan *et al.* (“patients deemed to be medically well enough for discharge but unable to leave because arrangements for the continuing care they need have not been finalised”) and to fill out an *ad hoc* form [10]. They were asked to make only clinical judgement, without taking into account the availability of rehabilitation, residential and domiciliary services, and thus reducing the risk of bias.

Over a study period of 15 days, medical and nursing staff completed daily a list of patients medically fit for discharge but still hospitalised. Patients with these characteristics will be denoted hereafter as delayed hospital discharges (DHDs). An index period of 15 consecutive days was chosen by the participating operative units between 30 April and 31 May 2011.^d^ The comparison group was derived from hospital discharge records (HDRs), and included all the other patients hospitalised in the same operative units during the index period.

### Data

Survey data collected for DHD patients included a list of 7 causes of delayed discharge selected by the Regional Work Group, whether the patients lived alone, and the index of dependence in Activities of Daily Living (ADL), that measures performance in the six functions of bathing, dressing, toileting, transferring, continence, and feeding. A score of 0 indicates no impairment, 2 indicates moderate impairment, and 4 or more indicates severe functional impairment [17].

Information retrieved from HDRs for DHD patients and for the comparison group included age, sex, elective or urgent admission, provision of intensive care, patient’s residence, primary diagnoses, procedures, and comorbidities.

Primary diagnosis was assessed at the index admission and comorbidities were assessed at the index admission and in the two previous years. Diagnoses were classified, using the enhanced ICD-9-CM Elixhauser algorithm [18], into 31 groups of diseases. We added to these groups of diseases dementia (codes 290.x, 294.1, 331.2) and femoral/shoulder fractures (codes 733.14, 733.15, 808.x, 810.x, 811.x, 812.0–812.1, 820.x, 821.x).

ICD-9-CM procedures were assigned, using a tool developed by the Agency for Healthcare Research and Quality [19], to one of four categories: minor diagnostic, minor therapeutic, major diagnostic and major therapeutic.

Hospitals were categorised as Local Health Authority Hospitals or Hospital Trusts. Operative units were categorised according to their discipline (general surgery, geriatrics, internal medicine, orthopaedics/traumatology, and long-term/rehabilitation). Long-term/rehabilitation units are acute hospital wards established in Emilia-Romagna in the late ‘90s that provide mostly nursing healthcare to patients that are still not self-sufficient and need especially nursing and rehabilitation care.

### Statistical analysis

One-day^e^ and two-week prevalence rates of DHD were calculated, with 95% confidence intervals based on the normal approximation to binomial distribution.

In order to identify the characteristics distinguishing DHDs from non-DHDs, a multilevel logistic regression analysis was carried out using individual data for all patients hospitalised on the first day of the index period, as it is not recommended to use period prevalence for case–control studies [20]. To take into account the hierarchical structure of our data, with patients nested into operative units, we fitted a two-level hierarchical logistic regression model, the first level being the patient level and the second the unit level. Because the operative units are in turn nested into Local Health Authority Hospitals or Hospital Trusts, we used a cluster robust estimator to obtain a reliable estimate of the standard errors for regression coefficients [21]. The patients’ demographic and clinical characteristics to be included in the multilevel model were identified in a preliminary stepwise logistic regression model (significance level of entry = 0.05; significance level of removal = 0.10). The second-level characteristics included in the model were the operative unit discipline and the type of hospital (Local Health Authority Hospital *vs.* Hospital Trust).

Multilevel logistic regression was carried out using the -gllamm- procedure of Stata software, version 12 (StataCorp. 2011. *Stata Statistical Software:* Release 12. College Station, TX: StataCorp LP).

### Ethics statement

This survey was part of the clinical governance activities of the Regional Healthcare Authority of Emilia-Romagna. These activities, aimed to promote the health of the population, are exempt from notification to the Local Ethics Committee because they consist of a review of patients’ charts (similarly to routine audit activities) and do not imply collection of additional data from patients. All patients signed a consent form at hospital admission for use of their data for administrative and research purposes, as is done routinely to comply with the privacy law, and no further consent was required. Survey data were linked to HDRs and then encrypted at the Regional Health Information System Office. This identifier does not allow to trace the patient’s identity and other sensitive data.

The study was carried out in conformity with the regulations on data management of the Regional Healthcare Authority of Emilia-Romagna, and with the Italian “Code of conduct and professional practice applying to processing of personal data for statistical and scientific purposes” (Art. 20–21, DL 196/2003) (http://www.garanteprivacy.it/web/guest/home/docweb/-/docweb-display/docweb/1115480, published in the Official Journal no. 190 of August 14, 2004) which explicitly exempts the need of ethical approval for encrypted data (Preamble #8) collected for scientific and healthcare purposes.

## Results

### Baseline characteristics and population case mix

Of the 256 participating operative units, 50 were general surgery (13.5%), 18 geriatrics (7.0%), 84 internal medicine (32.8%), 40 orthopaedics/traumatology (15.6%), and 64 long-term/rehabilitation (25.0%). In particular, we examined a total of 6,952 hospital beds: 1,130 in general surgery (16,3%), 562 in geriatrics (8.1%), 2,851 in internal medicine (41.0%), 1,093 in orthopaedics/traumatology (15.7%), and 1,316 in long-term/rehabilitation units (18.9%).

On the first day of the study period, 6,325 patients were hospitalised: 3,449 females (54.5%) and 2,876 males (45.5%), with a mean age of 74 years (Table 1). Most of the patients were living in the same province as that of the operative unit (89.2%). 79.5% of the patients had an urgent admission, 5.6% were provided intensive care and 85.3% had at least one comorbidity. Two thirds of the patients were hospitalised for therapeutic procedures—of these, 49.6% were minor and 50.4% major.

**Table 1:** Characteristics of the study sample

**Characteristics**	**All (*n*** **= 6,325)**	**DHD**	
		**No (*n*** **= 5,815)**	**Yes (*n*** **= 510)**
***Patients’ characteristics***			
**Age in years, mean [SD]**	73.9 [15.9]	73.5 [16.1]	78.4 [12.5]
**Sex (%)**			
Male	2,876 (45.5)	2,670 (45.9)	206 (40.4)
Female	3,449 (54.5)	3,145 (54.1)	304 (59.6)
**Admission status (%)**			
Elective	1,294 (20.5)	1,218 (20.9)	76 (14.9)
Urgent	5,031 (79.5)	4,597 (79.1)	434 (85.1)
**Patient’s residence (%)**			
Same province as that of the operative unit	5,640 (89.2)	5,177 (89.0)	463 (90.8)
Other province as that of the operative unit	269 (4.2)	253 (4.4)	16 (3.1)
Other region	387 (6.1)	361 (6.2)	26 (5.1)
Abroad	29 (0.5)	24 (0.4)	5 (1.0)
**Provision of intensive care (%)**			
No	5,972 (94.4)	5,510 (94.8)	462 (90.6)
Yes	353 (5.6)	305 (5.2)	48 (9.4)
**Procedure type (%)**			
Minor diagnostic	1,575 (24.9)	1,480 (25.5)	95 (18.6)
Minor therapeutic	2,103 (33.3)	1,892 (32.5)	211 (41.4)
Major diagnostic	63 (1.0)	59 (1.0)	4 (0.8)
Major therapeutic	2,133 (33.7)	1,961 (33.7)	172 (33.7)
None	451 (7.1)	423 (7.3)	28 (5.5)
Number of comorbidities^*^			
None	929 (14.7)	896 (15.4)	33 (6.5)
One	838 (13.2)	770 (13.2)	68 (13.3)
Two or more	4,558 (72.1)	4,149 (71.4)	409 (80.2)
***Operative unit and hospital characteristics***			
**Operative unit discipline (%)**			
General surgery	896 (14.2)	868 (14.9)	28 (5.5)
Geriatrics	543 (8.6)	508 (8.7)	35 (6.9)
Internal medicine	2,703 (42.7)	2,537 (43.6)	166 (32.6)
Orthopaedics/traumatology	907 (14.3)	820 (14.1)	87 (17.1)
Long-term/rehabilitation	1,276 (20.2)	1,082 (18.6)	194 (38.0)
**Type of hospital (%)**			
Local Health Authority Hospital	4,505 (71.2)	4,185 (72.0)	320 (67.2)
Hospital Trust	1,820 (28.8)	1,630 (28.0)	190 (32.8)

^*^Number of comorbidities retrieved at the index admission and in the two previous years.

DHD, delayed hospital discharge; SD, standard deviation; IQR, interquartile range.

42.7% of the patients were admitted to internal medicine units, 20.2% to long-term/rehabilitation, about 14% to general surgery and orthopaedics/traumatology, and 8.6% to geriatrics units. More than two thirds of the patients were admitted to Local Health Authority Hospitals (71.2%) (Table 1).

### Prevalence of DHD

The one-day prevalence of DHD was 8.1% (*n* = 510) (Table 2), while the two-week prevalence was 8.4% (*n* = 988) (Table 2). The highest one-day and two-week prevalence figures were found in long-term/rehabilitation units (15.2% e 16.8%, respectively), whereas the lowest prevalence was found in the general surgery units (3.1% and 3.4%) (Table 2). Over the entire study period, we found 8,797 DHD bed-days over a total of 92,822 bed-days (9.5%). On average, DHD patients occupied 590 hospital beds every day.

**Table 2:** **One-day and two-week prevalence of DHD with 95**% **confidence intervals**

**Operative unit discipline**	**DHDs (day 1)**	**DHDs (days 2–15)**	One-day prevalence (%)^*^	**Two-week prevalence (%)**
General surgery	28	69	3.1 (2.0–4.3)	3.4 (2.3–4.6)
Geriatrics	35	99	6.4 (4.4–8.5)	6.4 (4.3–8.4)
Internal medicine	166	424	6.1 (5.2–7.0)	6.3 (5.4–7.2)
Orthopaedics/traumatology	87	175	9.6 (7.7–11.5)	9.4 (7.5–11.3)
Long-term/rehabilitation	194	221	15.2 (13.2–17.2)	16.8 (14.6–18.9)
*Total*	*510*	*988*	*8.1 (7.4–8.7)*	*8.4 (7.7–9.0)*

^*^ One-day prevalence was calculated on the first day of the index period.

### DHD patients’ characteristics and causes of delay

Compared with non-DHDs, DHD patients were older (78.4 *vs.* 73.5 years, on average), experienced more frequently urgent admissions (85.1% *vs.* 79.1%), required more often intensive care (9.4% *vs.* 5.2%), and had more comorbidities (% of comorbid patients: 80.2% *vs.* 71.4%) (Table 1).

Causes of delay by operative unit discipline are presented in Table 3. Among a wide range of potential causes, waiting to access long-term/rehabilitation units or residential care homes were the most common (23.7% and 28.4%, respectively). Furthermore, 16.7% of the patients experienced a delay for family-related reasons, and 14.5% were waiting to be admitted to other rehabilitation services. Among DHD patients hospitalised in long-term/rehabilitation units, almost half were waiting to be transferred to residential care homes (45.3%). About 40% of DHDs in general surgery, geriatrics and internal medicine were associated with waiting for long-term/rehabilitation beds (37.0%, 40.0% and 42.1%, respectively).

**Table 3:** Causes of delay by operative unit discipline

**Cause of delay**	**Operative unit discipline**					
	**General surgery (*n*** **= 27)**	**Geriatrics (*n*** **= 35)**	**Internal medicine (*n*** **= 164)**	**Orthopaedics/traumatology (*n*** **= 87)**	**Long-term/rehabilitation (*n*** **= 190)**	**All (*n*** = 503)^*^
Awaiting acute/Rehabilitation hospital bed	8 (29.6)	4 (11.4)	27 (16.5)	20 (23.0)	14 (7.4)	73 (14.5)
Awaiting hospital bed in a long-term/Rehabilitation unit	10 (37.0)	14 (40.0)	69 (42.1)	26 (29.9)	0 (0.0)	119 (23.7)
Awaiting bed in a public/Private residential care home	3 (11.1)	10 (28.6)	25 (15.2)	19 (21.8)	86 (45.3)	143 (28.4)
No caregiver/Caregiver does not accept hospital discharge	6 (22.2)	2 (5.7)	26 (15.8)	7 (8.0)	43 (22.6)	84 (16.7)
House inappropriate for home care/Patient resides outside the region	0 (0.0)	0 (0.0)	2 (1.2)	5 (5.7)	16 (8.4)	23 (4.6)
Awaiting home care/Unavailability of medical devices and medical equipments	0 (0.0)	5 (14.3)	7 (4.3)	5 (5.7)	15 (7.9)	32 (6.4)
Delay in referral for social service assistance or general inefficiency in sheltered discharge procedure/Other	0 (0.0)	0 (0.0)	8 (4.9)	5 (5.7)	16 (8.4)	29 (5.8)

^*^Patients with unknown cause of delay were not included (*n* =7).

One out of four DHD patients lived alone and 57.6% had full function or moderate impairment in daily activities (ADL ≤ 2).

### Predictors of DHD

Results of multilevel analysis for all patients hospitalised on the first day of the index period (*n* = 6,325), are presented in Table 4. Patients hospitalised in long-term/rehabilitation units, as well as in orthopaedics/traumatology units, were significantly more likely to be DHDs compared with patients hospitalised in general surgery units [adj. OR (95% CI) = 5.65 (3.02–10.56) and 3.92 (2.24–6.85), respectively]. Also patients in internal medicine units were more likely to be DHDs [adj. OR (95% CI) = 1.78 (1.01–3.14)] than patients hospitalised in surgical units. Moreover, compared with Local Health Authority Hospitals, being hospitalised in Hospital Trusts was associated with a higher likelihood of DHD [adj. OR (95% CI) = 1.69 (1.12–2.54)].

**Table 4:** Multilevel logistic regression analysis: adjusted odds ratios of DHD as a function of patients’ characteristics, operative unit discipline and type of hospital

	**Adj. OR**	***p*-value**	**95%CI**
***Patients’ characteristics***			
**Age (decades)**	1.24	<0.001	1.16–1.33
**Provision of intensive care**			
No	1		
Yes	1.87	0.026	1.08–3.23
**Primary diagnosis**			
Dementia	3.47	0.001	1.65–7.28
Tumour	1.71	0.007	1.16–2.51
Femoral/Shoulder fractures	1.52	0.013	1.09–2.11
**Number of comorbidities**			
None	1		
One	1.92	0.006	1.21–3.04
Two or more	2.03	<0.001	1.45–2.84
***Operative unit and hospital characteristics***			
**Operative unit discipline**			
General surgery	1		
Geriatrics	1.38	0.299	0.75–2.54
Internal medicine	1.78	0.045	1.01–3.14
Orthopaedics/Traumatology	3.92	<0.001	2.24–6.85
Long-term/Rehabilitation	5.65	<0.001	3.02–10.56
**Type of hospital**			
Local Health Authority Hospital	1		
Hospital Trust	1.69	0.012	1.12–2.54

OR, odds ratio; CI, confidence interval.

Patient-level characteristics associated with a higher likelihood of DHD were: age in decades [adj. OR (95% CI) = 1.24 (1.16–1.33)], provision of intensive care [adj. OR (95% CI) = 1.87 (1.08–3.23)], specific primary diagnoses – the most relevant being dementia, tumours and femoral/shoulder fractures, − and having at least one comorbidity [adj. OR (95% CI) = 1.92 (1.21–3.04)] or more than one [adj. OR (95% CI) = 2.03 (1.45–2.84)].

## Discussion

In Emilia-Romagna region hospitals, during the index period, 8% of patients experienced a delay in discharge, which amounts to about 590 (10%) beds per day occupied inappropriately. This figure is close to the lower boundaries of the prevalence ranges reported in two systematic reviews on inappropriate use of acute care beds (15%–50% [4] and 8%–66% [14]).

Measures to optimise hospital care resources have been introduced in the region in the last fifteen years, starting from the implementation of hospital networks based on the hub and spoke model, that have been followed more recently by interventions aimed to improve patient flow logistic according to clinical severity and complexity of care. Strategies for discharge planning have also been implemented, supported by nurses and other staff in the role of discharge coordinators. These policies and strategies had the objective to foster care coordination for older people with complex needs and to reduce healthcare costs. It is possible that the low prevalence of DHD in the region is partially accounted by these organisational efforts, in line with a recent study [12] showing that medical staff involvement and discharge planning significantly reduced DHDs in a teaching hospital.

However, the need to cut hospital budget and reduce the number of bed requires further efforts to rationalise the use of beds. In this sense, the study of the delayed discharge phenomenon, and specifically of its organisational and individual determinants, may help stakeholders to develop informed strategies and policies.

In the present study, a remarkable variability was observed in the prevalence of DHDs among the five disciplines considered, from 3% in general surgery to 15% in long-term/rehabilitation units, that proved to be the main bottleneck. In these units, almost half of DHD patients (45.3%) (Table 3) were waiting to be transferred to residential facilities and 22.6% did not have a caregiver at home. Conversely, 42.1% of DHD patients in internal medicine and 40.0% in geriatric wards were waiting for a bed in long-term/rehabilitation units. Our results are consistent with Seymour *et al.* and Coid *et al.*[22,23], who identified a major risk of DHD for medical specialties and very few bed-blocking patients in surgical wards. Several factors may account for this observation. The younger age of patients treated in surgical wards indicates that this patient group is less likely to pose the multiplicity of problems which characterise elderly patients. Another explanation could be that surgery units discharge patients or transfer them elsewhere as soon as possible to make their bed available [11].

As to the demographic and clinical determinants of DHDs, we found that increasing age, the number of comorbidities, a primary diagnosis of dementia, fracture or tumour, and the provision of intensive care were associated with an increased likelihood of DHD. Specifically, each decade of life increased the risk of DHD by 24% (*p* < 0.001). These results altogether support the evidence that elderly patients are more prone to have protracted hospital stay [3,24] because they present with multimorbidity or with specific problems like cognitive impairment or orthopaedic conditions that may require rehabilitation, domiciliary services or some form of institutional care that may be not immediately available at discharge [25,26].

Of note, we found that Hospital Trusts were more likely to have DHD patients than Local Health Authority Hospitals. A possible explanation is that Hospital Trusts have weaker links with the primary care sector, that is managed by Local Health Authorities.

The evidence provided by our study supports the need to set up service delivery models meant to reduce the hospital stay (especially for older patients hospitalised in long-term/rehabilitation units or waiting to access them) through the provision of enhanced health and social care arrangements. In Australia, Canada and the US, a number of programmes were implemented in the hospital units, including the Acute Care for Elders (ACE) [27], the Hospital Elder Life Program (HELP) [28] and orthopaedic-geriatric medicine cocare [29]. These programmes were associated with significant reductions in morbidity and mortality, and increases in optimal postoperative care [28,29], and proved to prevent significantly functional decline, reduce cost of care and length of hospital stay, and increase home discharge [30]. However, complex care models such as the ACE programme (including patient-centred care, frequent medical review, early rehabilitation, and early discharge planning) are difficult to implement in acute hospital wards on a routine basis [31].

In the community setting, service delivery models aimed to facilitate rehabilitation and hospital discharge, or more holistically care close to home, have been denominated in various ways like “hospital at home”, “nursing led in-patient units”, “general practitioner run community hospitals”, “intermediate care in nursing homes” and “community care centres” [32,33], and in the UK they have been named “intermediate care”. A recent realist review has been published by Pearson *et al.*[34] with the aim to build up a conceptual framework for intermediate care to investigate under which circumstances it is likely to be feasible and effective and, most important, cost-effective. However, evidence on this topic is still lacking.

One strength of the present study is that it included a large number of units from different medical specialties, and a relatively large number of patients. Despite this significant strength, this study also has an important limitation. Patients were considered eligible for inclusion in the study at any point during their hospital stay: this could have accounted for the low prevalence of DHD and confounded results, as patients who are at the end of their stay are more likely to be designated DHDs compared with those that are newly admitted. However, since the one-day and two-week prevalence rates reported in the present study are very similar, the cross-sectional assessment did not lead to an underestimation of the phenomenon.

## Conclusions

The results of our study highlight the need to face the DHD problem with major organisational innovations, with a special focus on the ageing of the population and epidemiological trends. Organisational changes imply new ways of managing emerging clusters of patients whose needs are not efficiently or effectively met by traditional organisation models and services.

## Endnotes

^a^The 24 non-participating operative units were already involved in other research projects and lacked the means to train nurses and physicians to conduct the survey.

^b^Local Health Authorities are vertically integrated organisations funded by the region through a capitated budget and responsible for a wide range of hospitals and community services in geographical areas with populations ranging 60,000 to 1,000,000 inhabitants.

^c^Hospital Trusts are major hospitals providing mainly tertiary care, and their legal status is similar to that of the British trust hospitals. Some of them are affiliated with a medical school (teaching hospitals).

^d^May is far from flu season and summer period (when bed availability and services are reduced for holiday), and might therefore be considered as representative of the average NHS bed occupancy over the year. The choice of 15 consecutive days was arbitrary and based on convenience.

^e^One-day prevalence was calculated on the first day of the index period.

## Abbreviations

NHS: National healthcare system; DHD: Delayed hospital discharge; HDR: Hospital discharge record; ADL: Index of dependence in Activities of daily living; ICD-9-CM: International classification of diseases (clinical modification, ninth revision); SD: Standard deviation; IQR: Interquartile range; OR: Odds ratio; CI: Confidence interval; ACE: Acute care for elders; HELP: Hospital elder life program.

## Competing interests

The authors declare no competing interests.

## Authors’ contributions

JL conceived the statistical methodology, performed the statistical analysis and drafted the manuscript; MM conceived the study design and contributed to the final writing of the paper; PR conceived the statistical methodology and drafted the manuscript; ED participated in the study design and contributed to the final writing of the paper; MV provided data sources and performed the statistical analysis; CR critically revised the draft and contributed to the final writing of the paper; ET took responsibility for the integrity of the data and performed the statistical analysis; TC participated in the study design and critically revised the draft; MPF contributed to the conception of this paper, conceived the study design and drafted the manuscript. All authors read and approved the final version of the manuscript.

## Pre-publication history

The pre-publication history for this paper can be accessed here:

http://www.biomedcentral.com/1472-6963/14/128/prepub
